# Syphilis, with Special Reference to the Relations of the Dental Profession to the Disease

**Published:** 1895-09

**Authors:** Henry A. Pulsford


### SYPHILIS, WITH SPECIAL REFERENCE TO THE RELATIONS OF THE DENTAL PROFESSION TO THE DISEASE.[1]

BY HENRY A. PULSFORD, M.D.

Many, if not most, of those present here this evening would probably assert quite confidently that they have never had a syphilitic patient in their operating-chairs. Yet I venture to state that there are very few busy dentists who do not unwittingly treat at least one syphilitic in the course of each year of practice. The disease is an extremely common one. In the practise of skin-specialists about one-tenth of all the cases seen are forms of syphilis, and in order of frequency it stands third upon the list of skin-diseases. As it is contracted by the rich and intelligent almost as often as by the poor and ignorant, you are always liable, no matter among what class you practise, to be called upon to treat some person who carries in his mouth the poison of the disease. If he happen to infect you, it means at the very least two years' abstinence from professional work, to say nothing of the social and domestic privations such as infection necessarily imposes. If, through you or your instruments, he infect one or more of your patients, it means great and possibly irreparable injury to you and to your practice. It should therefore be of the utmost interest to you as dentists to have accurate information about this disease; and it is in the hope of adding to your stock of knowledge of the subject that I venture to present this paper to-night.

Syphilis is a chronic infectious disease, produced by a specific poison, which, in all probability, is a bacillus closely resembling that of tuberculosis. Inoculation with this virus causes a speedy saturation of the whole system with the disease, and, except for the transient local reaction at the site of the inoculation, the malady in its subsequent course shows all the characteristics of a general, constitutional disease.

---

[1] Read before the Central Dental Association of Northern New Jersey, June 10, 1895.

Syphilis may be acquired in several different ways: The germs may be deposited upon an abrasion, wound, or other solution of continuity of skin or mucous membrane. In the case of a woman impregnated by a syphilitic, they may enter the system through the placental circulation. The child begotten by a syphilitic father is usually syphilitic, and a syphilitic mother generally brings into the world an infant who inherits her disease.

The germs of syphilis are present in the discharges from all the early eruptions and ulcerations, and in the blood, lymph, semen, and ova of those passing through an active stage of the disease. Infection through the unbroken epidermis, either of the skin or mucous membrane, is impossible. "The venereal disease" *par excellence*, syphilis, is usually acquired during sexual intercourse. Liability to the disease is practically universal, the only known immunity being that conferred by the disease itself, which protects the individual against a second attack. Very few prostitutes escape it, the majority of them being infected in the course of the first two years of their loose life. Hence it is most frequent where prostitution is rife, or where promiscuous sexual relations prevail.

After the expiration of a more or less definite period of incubation, infection with syphilis is followed by the development, at the site of the inoculation, of the "initial lesion" of the disease. This is first seen usually as a hard, painless papule, which soon breaks down into a shallow ulcer, secreting a scanty discharge, and causing considerable induration of the surrounding tissues. Such are the typical characteristics of the dreaded "hard chancre." The formation of this apparently trifling ulcer is a sign that the whole system is already hopelessly infected, and no local treatment, however heroic, can prevent the further development of the disease. Coincident with the appearance of this ulcer occurs the enlargement of the neighboring lymphatic glands, and within a short time a similar condition of all the glands of the body, together with the establishment of certain moderate constitutional symptoms, furnishes abundant proofs that the disease has indeed become general.

The eruptive period of syphilis follows the appearance of the initial lesion in from three to twelve weeks. It is ushered in by moderate fever, headache, pains in the joints and bones, and much general malaise. These symptoms are immediately followed by the appearance of an eruption occurring usually simultaneously on the skin and mucous membranes. The first eruption is commonly very superficial, consisting of spots of localized hyperæmia, like those of measles. It soon fades away, to be followed in the course of a few weeks by a second eruption, generally of a papular form. This in turn disappears, giving place, in a longer or shorter time, to still another and severer form of eruption. Thus the disease goes on, gathering strength as it develops, until the climax of the eruptive period is reached in a general eruption of pustules or boils, which may be so severe that small-pox, as its name implies, is trifling in comparison.

In the course of the eruptive period, which often lasts two or three years, there also occur many other manifestations of the disease. The general health fails, there is anæmia and loss of flesh and strength, there are ulcerations of the various mucous membranes, the hair falls out, certain diseased conditions of the eyes and ears may develop, and the bones and joints may become affected.

After the second or third year there is often a decided lull in this storm of symptoms. Sooner or later, however, the disease again makes its presence and power felt by new and severer manifestations. These, the so-called *tertiary* or *late* lesions of syphilis, differ decidedly from those which occur in the eruptive stage. They are rarely general in distribution, being confined to a limited portion of the body; they are extremely chronic, each distinct attack lasting for months or years; they often spread slowly, in a serpiginous manner, from the point at which they start; they rarely secrete infectious discharges; they often cause extensive destruction of tissue, which, occurring in important organs, may occasion a fatal issue; they are more likely to attack deep-seated structures and vital organs than are the lesions of the eruptive period.

Fortunately for the many persons who contract syphilis, it is no more necessary for them to experience all the possible lesions of the disease than it is for a guest at a fashionable hotel to partake of every delicacy on the bill of fare. The majority of syphilitics have, besides their chancre, two or three moderately severe eruptions, a partial loss of hair, several ulcerations in the mouth or throat, and one of the slight or moderately severe late lesions. When properly treated from the first, the disease usually runs a rapid and benign course, leaving its victim somewhat impaired in health, to be sure, but only exceptionally a physical wreck or chronic invalid.

The foregoing brief sketch will give you some idea of syphilis as it is seen by physicians. Dentists, in the practice of their profession, naturally see much less of the disease; but that they have occasional opportunities for observing many of its characteristic lesions there can be no question. On the lips they may possibly see the initial lesion; for, although of rare occurrence, chancre of the lip is the most common of all extragenital chancres. More rarely still they may meet with a chancre of the tongue, of the tonsil, or of some part of the face. The characteristics of all these lesions are similar in whatever locality they occur. The ulcer may be as small as that of the ordinary chapped lip, or it may be as large as an inch in diameter. The most constant characteristics of the syphilitic chancre are the parchment like induration of the underlying tissues and the painless enlargement of the nearest lymphatic glands.

In the eruptive period the dentist may see such of the eruptions as occur on the head and neck, the ulcerations and eruptions in the mouth and nose, and the diseases of the eyes. One of the most typical of the facial eruptions is the so-called "corona veneris," a well-marked semicircle of large papules crowning the forehead just below the line of the hair. The presence of this eruption, with a decided thinning of the hair on the sides of the head, is pretty strong proof of syphilis. Another characteristic manifestation of syphilis which a dentist may see is the "angina syphilitica," or syphilitic sore throat. It is a vivid, raw, ham-colored hyperæmia of the pharynx, tonsils, and soft palate, which is limited by a definite margin, especially well marked just above the uvula and the free border of the soft palate. Portions of the inflamed areas may be coated with a grayish layer of dead epidermis, or even superficially ulcerated. The most common locations for the oral ulcerations of this stage of the disease are the angles of the lips and the tip and margins of the tongue. Associated with these ulcerations, or occurring independently of them, may often be seen red spots, generally circular in shape, having sharply-defined edges, and flecked with a grayish deposit of epidermis. All syphilitic lesions of the mouth seem more apt to occur upon parts subject to continual irritation, such as that produced by a cigar or pipe, by a plate for artificial teeth, or by the roughened edges of broken or carious teeth. Clean and well-kept mouths are less liable to these complications than are foul and neglected ones.

The most common oral lesions of late syphilis are perforations and destruction of the hard and soft palates. They are almost always accompanied by a very offensive nasal catarrh. Deep ulcerations of the tongue and tonsils may also occur in this stage. It is always satisfactory to remember that the discharges from all these destructive ulcerations are usually quite harmless, and incapable of producing the disease in another person.

Not the least of the dangers of syphilis are the diagnostic difficulties which it offers even to the experienced physician. If it were possible to give you an infallible rule by which you might detect the presence of the disease, it would, as far as you are concerned, be robbed of many of its perils. Unfortunately, however, there is no such rule. The most innocent-looking lesions sometimes prove to be syphilitic, and the most suspicious in appearance frequently turn out to be harmless. Even physicians who are familiar not only with syphilis, but also with the many diseased conditions with which it may be confused, are occasionally mistaken in their diagnoses and misled by appearances. Therefore, as a dentist is not necessarily an experienced syphilographer, he will scarcely attempt to make his own diagnosis of an unusual oral condition, but will in all such cases get the opinion of a competent physician.

A dentist finding himself face to face with a syphilitic patient who needs dental treatment has two courses open to him: he may refuse to take the case, either setting him adrift or turning him over to a less fastidious colleague, or he may accept and treat him just as he would any other case that comes to him. Now, I have neither the desire nor the presumption to lay down the law as to the conduct of the members of your profession, either under these or any other circumstances. I hold that every man, whether he be a dentist or a physician, has a perfect right to use his own judgment as to what cases he ought to treat and what cases he ought not to treat. That is his business. You can scarcely accuse me of meddlesome interference, however, if I merely place before you fairly and squarely the *pros* and *cons* of this matter.

In the first place, you may urge that you are justified in refusing to treat syphilitics simply and solely on account of the personal risk incurred. That is perfectly true, and the argument is unanswerable. But it is well to bear in mind that you do not avoid this risk altogether by refusing to treat such persons as you know are syphilitic. As a class, syphilitics need a great deal of dental treatment, and they generally contrive to get it sooner or later. Some do so innocently, being ignorant of the nature of their disease and of the risk they are imposing upon the dentist. Others are aware that they have syphilis, but do not know that they have infecting lesions in their mouths. Still others know all about their condition and its dangers, but do not care how much injury they inflict upon their fellows; they will shamelessly lie about themselves, and, in order to hoodwink the dentist, will try to mitigate and conceal their symptoms, as prostitutes do when syphilis threatens to ruin their business. It is thus quite evident that a dentist escapes only a part of the risk of infection by refusing to treat cases which he knows are syphilitic; for in the recognized cases of the disease he can, by taking proper precautions, easily avoid infection; but where he does not suspect it he will naturally be more or less off his guard.

That a dentist ought to refuse to treat syphilitics from fear of infecting other patients is a good pretext, but can hardly be made a substantial ground for argument; for, as I have already said, in speaking of the personal risk, so also may I say of the risk to your patients, that it cannot be altogether avoided by refusing such cases. In the second place, in these days of wide and growing knowledge of germs and germ-diseases, when we all know that the mouth is a perfect hot-bed of bacteria, a dentist is surely not guiltless if he fail to take the simple precautions which will make it almost impossible for him to carry any germs whatsoever from the mouth of one patient to that of another. A dentist who always thoroughly cleanses his hands and sterilizes his instruments before working upon each patient runs very little danger of transmitting syphilis or any other infectious disease. I will not deny, however, that it would be the part of wisdom for dentists who knowingly do much work among syphilitics to have, as many physicians have, a set of instruments for use only upon such cases.

A third argument which I have heard urged against the treatment of syphilitics is the "social-morality" argument. Some "good" people argue that, as syphilis is invariably contracted through gross immorality, it is the duty of every true Christian, whether he be dentist, doctor, or preacher, to prevent the wretched sufferer from obtaining the slightest comfort or relief under this visitation of a justly incensed Providence. Of course, you all know that this is nonsense. A syphilitic is very rarely a more grossly wicked man than the majority of his fellows, unless he has been made to feel that he is a social outcast. I have known young men who acquired the disease in their very first step aside from the path of virtue; I have known good women—wives and mothers—who have been infected by vicious husbands; and every one of you know men who, in spite of long lives of licentiousness, have escaped this scourge.

We of the healing professions will do well if we let theology and casuistry alone. It is not our business to punish men and women for doing evil, but, as far as in us lies, to cure and to prevent disease. Even if we should all unite in refusing to treat these troubles, we should not only fail to advance the standard of social morality, but would inflict untold suffering upon countless innocent persons. There is too much syphilis, too much gonorrhœa in the world.

These maladies are not weeding out the vicious from society; their prevalence is not improving the human race; on the contrary, it is making it worse. Constitutions are undermined, individuals are weakened physically, mentally, and morally, and their offsprings are weaklings, unable to resist either disease or temptations. Let the clergy do their part in preaching sexual purity to the people; but let us also do our part, which is the curing of these diseases and the stamping them out of existence. With reference to venereal diseases, it always has been and always will be my practice to make treatment and relief as easy and as accessible as possible for all the afflicted. I do this not from any feeling of weak sentimentality for the sufferers, but for the benefit of humanity at large. Every syphilitic is a centre of infection, and carries with him the possibilities of infinite mischief to his fellows. If our laws permitted it, we should do well to incarcerate such persons, and subject them to proper isolation and treatment until they were cured; but as we are too fond of what we call "personal liberty" to do this, let us at least do our best to cure them,—and that, too, as speedily as may be.

In the scientific treatment of syphilis the dentist may, if he will, play a very helpful part. If all things were done as they should be done, the first step in the treatment of a syphilitic would be to send him to a dentist to have his teeth and mouth put in as good a condition as possible. This can in most cases be done before there are any infectious lesions in the mouth. Then, when the physician comes to put his patient through the courses of mercurial treatment, which are so essential to a thorough cure, there will be little danger of the occurrence of salivation or stomatitis, which check treatment, and make the remedy seem as bad as the disease. When the ulcerations attack the mucous membranes, there will be no severe lesions in the mouth from carious, jagged teeth. In short, if a syphilitic has a mouth filled with smooth, clean, healthy teeth, it is easy to cure him; but if his teeth are dirty, ragged, and carious, his cure is always difficult, and sometimes impossible.

If dentists as a class refuse to do their small but important part in the treatment of syphilis, they are working against those who are trying to exterminate this horrible disease. If they wish to help us who are doing our best to rid society of this scourge, they will put to one side all thought of personal risk, and, confident in their knowledge and skill, will cheerfully undertake and accomplish their share in the good work.

